# Cultural interplay in end-of-life care decisions: comparing advance directive beliefs and preferences among adults in the U.S. and Taiwan

**DOI:** 10.1186/s12904-025-01736-z

**Published:** 2025-04-15

**Authors:** Duan-Rung Chen, Yuchi Young, Ashley Shayya, Taylor Perre, Thomas O’Grady

**Affiliations:** 1 Institute of Health Behaviors and Community Sciences, National Taiwan University College of Public Health, Room 636, No. 17, Xu-Zhou Road, Taipei, TW Taiwan; 2 Department of Health Policy, Management, and Behavior, University at Albany, College of Integrated Health Sciences, Rensselaer, NY US; 3 Center for Human Services Research, University at Albany, Albany, NY US; 4 Home Care Association of New York State, Albany, NY US; 5 Department of Epidemiology and Biostatistics, University at Albany, College of Integrated Health Sciences, Rensselaer, NY US; 6 Population Health Research Center, College of Public Health, National Taiwan University, Taipei, Taiwan; 7 Department of Health Policy, Management & Behavior, College of Integrated Health Sciences, University at Albany, 1 University Place, Room 171, Rensselaer, NY 12144 USA

**Keywords:** Advance directives, End-of-life care, Cross-cultural comparison, American and Taiwanese adults

## Abstract

**Background:**

Advance directives are essential to advance care planning, allowing individuals to document their end-of-life care preferences in a living, legally binding document. Cultural factors such as collectivism and family values can shape beliefs and preferences toward advance directives.

**Aim:**

This study compared beliefs and preferences toward advance directives between American and Taiwanese adults.

**Design:**

Cross-sectional survey. A multivariate logistic regression was used to quantify the differences between groups.

**Setting/participants:**

Age 18 + residing in the U.S. (*n* = 166) and Taiwan (*n* = 186).

**Results:**

Compared to the Taiwanese sample, the U.S. sample had more males (37% vs. 21%), more individuals with a graduate education (53% vs. 22%), and fewer single/unmarried participants (38.9% vs. 46.4%). In the multivariate logistic regressions, adults in Taiwan were 2.5 times more likely to value the importance of having an advance directive (aOR 2.5; 95% CI 1.27–5.12), 7.75 times more open to end-of-life care discussions (aOR 7.75; 95% CI 2.03–29.50), and 1.7 times more likely to allow family and loved ones make medical treatment and care decisions during hospitalization for a serious illness on their behalf (aOR = 1.73; 95% CI 1.08–2.78) compared to adults in the U.S. However, adults in Taiwan were less confident that their loved ones’ decisions would align with their personal preferences (aOR = 0.28; 95% CI 0.16–0.47).

**Conclusion:**

Adults in Taiwan place significant importance on advance directives and demonstrate a greater propensity to engage in end-of-life discussions. They also appear more willing than adults in the U.S. to delegate healthcare decisions to their loved ones. Paradoxically, however, they express concerns about whether these decisions align with their personal preferences, a discrepancy likely influenced by cultural values of filial piety and collectivism in Taiwan.

## Introduction

Advance directives are essential to advance care planning, allowing individuals to document their end-of-life care preferences in a living, legally binding document [1]. Advance directives may include a Living Will and Medical Power of Attorney, among others. They promote patient autonomy, enabling physicians to provide treatment and care that aligns with patients’ beliefs when patients can no longer make decisions [2]. Advance care planning and advance directives have been associated with increased patient and clinician satisfaction with communication [3, 4], reduced distress for surrogate decision-makers and clinicians [3], improved end-of-life care quality [4, 5], reduced end-of-life Medicare spending [6], and lower out-of-pocket costs [7, 8]. Despite these benefits, only 37% of American adults [9] and less than 1% of Taiwanese adults [10] have completed advance directives.

Advance directives and advance care planning have been prevalent in Western nations since the 1967 creation of the first directive by the Euthanasia Society of America [11]. U.S. states passed related legislation in the 1970s, culminating in the 1990 Patient Self-Determination Act [11, 12]. In contrast, advance care planning was not legally adopted in Asian countries until recently. Taiwan enacted such legislation with the 2016 Patient Right to Autonomy Act, which took effect in 2019 [13, 14]. 

Cultural factors strongly influence one’s beliefs and attitudes toward end-of-life care and advance care planning [13, 15–17]. Western approaches prioritize patient autonomy in end-of-life care decisions [18]. In Eastern Asian countries, tradition and culture are strongly influenced and shaped by Confucius’ teachings [13, 17, 19]. Confucius’ teachings focus on morality, social relationships, justice, and sincerity, each providing a solid framework for many Taiwanese values and beliefs [19]. Moreover, Confucian values of filial piety and familism/collectivism, emphasize family-led decision-making [13, 17, 19, 20]. 

Given these cultural influences, family-led medical decision-making is not uncommon in Taiwan, with family members playing a prominent role in the process [13, 20]. It is customary for adult children to make decisions for their older parents, seen as a part of respect and filial piety [20]. Confucian ideals such as filial piety and familism/collectivism can deter advance directive completion in Taiwan. Some adults in Taiwan fear that engaging in advance care planning and completing an advance directive may lead to family conflicts, lack of support from their relatives, or a perceived inability to make independent medical decisions [17, 21, 22]. Discussing death is often considered taboo, further limiting communication about advance care planning and advance directives [13, 17, 20, 21]. However, a recent study confirmed that adults in Taiwan who value “end-of-life pro-individualism” are more likely to complete advance directives [23] than those who value pro-collectivism/familism.

Our study examined and compared the beliefs, experiences, and preferences regarding advance care planning and directives among adults in Taiwan and the U.S., mainly in New York State. We hypothesized that cultural differences may underlie the varying attitudes toward advance directives between adults in the U.S. and Taiwan. Specifically, we anticipated that adults in Taiwan might exhibit greater reluctance to engage in these practices due to cultural principles rooted in Confucian teachings, which prioritize filial piety, familial harmony, and collective decision-making. In contrast, adults in the U.S., mainly in New York State, may emphasize individual autonomy and personal choice and contribute to a more proactive approach toward advance care planning and advance directive completion.

## Methods

A cross-sectional survey was conducted to collect data. The study received an expedited Institutional Review Board (IRB) review and approval from the University at Albany (IRB00000589; Protocol #21E071) and the National Taiwan University Research Ethics Committee (202205HM116), as it posed a minimal risk, included no sensitive questions, and did not involve vulnerable populations. Participants were not exposed to any risks beyond those encountered in daily life.

### Participant recruitment

In the U.S., participants aged 18 or older (*n* = 162) and able to sign informed consent forms were recruited using snowball sampling through personal contacts, including collaborators, colleagues, friends, and churchgoers. Participants were asked to invite others from their networks to complete the survey. All participants were required to sign an informed consent form and could withdraw at any time. Data were collected from March to August 2022.

In Taiwan, 941 participants were similarly recruited using snowball sampling via personal contacts between August and December 2022. Participants (aged 18+) signed informed consent forms before completing the survey. A small gift valued at NT$200 (approximately US$6.25) was provided for participation. The Taiwan sample (*n* = 168) was age-matched to the U.S. sample to minimize bias due to sample size differences and ensure better comparability.

### Survey instrument

Data were collected using structured questionnaires. Participants in the U.S. completed self-administered printed surveys, which took approximately 45–60 min, whereas, in Taiwan, the survey was conducted online. Both surveys assessed beliefs and preferences regarding advance directives. The U.S. questionnaire, which assessed beliefs, preferences, experiences, and knowledge of advance directives, was initially developed in English for the U.S. population and later translated into Traditional Chinese. The survey was adapted to fit the local context in Taiwan.

### Study variables

#### Dependent variables

Four questions from the questionnaire regarding beliefs and preferences toward advance directives were used as dependent variables (yes/no):

1. Perceived importance of preparing an advance directive (*At your current stage of life, do you believe it is important to prepare an advance directive for yourself*).
2. Willingness to discuss end-of-life care (*In general, are you willing to discuss end-of-life care for yourself and your significant others*).
3. Willingness to let family and loved ones make health care or end-of-life care decisions during hospitalization for a serious illness (“*Would you prefer to let to let your family and loved ones make your health care/end-of-life care decisions for you during your hospitalization for a serious Illness?”*).
4. Belief that medical treatment/care options decisions between self and family would be consistent (“*Do you believe decisions about medical treatment/care options made by your family and loved ones on your behalf would be consistent with your wishes?”)*.

#### Covariates

##### Sociodemographics

Selected sociodemographic variables included age (young adults aged 18–30, adults aged 30–64, older adults aged 65+), gender (male/female), marital status (single, married/domestic partner, divorced/separated, widowed), educational attainment (junior high school/middle school and below, high school and equivalent, college, graduate school or higher), and current employment status (yes/no). Due to differences between the English and Taiwanese surveys, some sociodemographic response options were merged for consistency (e.g., the English survey had separate options for “separated” and “divorced,” while the Taiwanese version combined them into a single category: “separated/divorced.” Consequently, the English survey responses for “separated” and “divorced” were merged to match the Taiwanese format). Participants’ previous experience with end-of-life care planning was assessed by asking respondents whether people close to them had engaged in end-of-life care planning (yes/no).

### Data analysis

The Taiwanese sample was age-matched to the U.S. sample for comparability. Bivariate analyses were conducted to examine the relationships between key study variables, including Chi-Square tests for associations between categorical variables and t-tests for comparing means between two groups. Multivariate logistic regressions assessed differences in beliefs and preferences, controlling for covariates. Analyses were conducted using SPSS 25.

## Results

Three hundred forty-eight respondents engaged, with 162 from the U.S. and 186 from Taiwan. Table 1 presents the bivariate analysis of sociodemographic characteristics and advance directive beliefs and preferences. There was no significant difference in age distribution between the two groups (*p* = 0.26), as age was used to match the populations for comparability. However, a significant difference (*p* < 0.01) was found in gender distribution, with 37% of participants in the U.S. being male compared to only 21% of participants in Taiwan. Educational attainment showed notable contrasts: the proportion of college-educated participants was significantly higher in the Taiwan sample (63.4%) than in the U.S. sample (31.5%; *p* < 0.01). Conversely, in the U.S., a significantly higher proportion of participants attained a graduate-level education (53.1%) than in Taiwan (22.0%). There was also variability in marital status, with a higher proportion of single participants in Taiwan (46.2%) compared to the U.S. (38.9%) (*p* = 0.05). Regarding employment, 58.0% of the participants in the U.S. were employed full-time, compared to 49.5% in Taiwan, a marginally significant difference (*p* = 0.07).

Notable differences in experiences with advance directives were observed between the two countries. When asked, “Has anyone close to you had engaged in end-of-life care planning?” a higher proportion of U.S. participants (53.7%) reported engagement compared to Taiwanese participants (40.3%, *p* < 0.01). Significant differences also emerged in beliefs and preferences toward advance directives. While most participants acknowledged the importance of preparing advance directives, participants in Taiwan reported significantly higher recognition (89.3%) than participants in the U.S. (79.6%, *p* = 0.01). Willingness to discuss end-of-life care was also higher in the Taiwan sample (98.4%) compared to the U.S. sample (90.7%, *p* < 0.01). Moreover, more participants in Taiwan (59.1%) were willing to let family and loved ones make health care/end-of-life care decisions during hospitalization for a serious illness on their behalf than participants in the U.S. (46.3%, *p* = 0.01). However, the belief that medical treatment/care options decisions between self and family would be consistent was significantly higher among participants in the U.S. (78.4%) than those participants recruited in Taiwan (50.5%, *p* < 0.01).

Table 2 presents a multivariate logistic regression analysis examining the differences in beliefs and preferences toward advance directives between participants recruited in Taiwan and those recruited in the U.S. while adjusting for age, gender, marital status, level of education, employment status, and whether respondents have anyone close engaged in “advance directives”? Using participants in the U.S. as the reference group, participants in Taiwan had significantly higher odds (aOR = 2.55; 95% CI 1.27–5.12, *p* < 0.01 ) of believing that preparing an advance directive was important (Model 1). Participants in Taiwan were also much more willing to discuss end-of-life care (aOR = 7.75; 95% CI 2.03–29.50, *p* < 0.01) than their American counterparts (Model 2). Participants in Taiwan were also more likely to let family and loved ones make health care or end-of-life care decisions during hospitalization for a serious illness on their behalf than participants in the U.S. (aOR = 1.73; 95% CI: 1.08–2.78, *p* < 0.05) (Model 3). Lastly, participants in Taiwan were less confident (aOR = 0.28; 95% CI 0.16–0.47, *p* < 0.001) that decisions made by their family regarding medical treatment/care options decisions would be consistent with their wishes compared to those participants in the U.S. (Model 4).

### Covariates

College-educated individuals are 54% less likely, and those with graduate education are 68% less likely to defer healthcare decisions to family than those with a high school education or less (Model 3). The findings indicate that higher education is associated with a stronger preference for individual autonomy in healthcare decisions. Those with more education may be more informed about medical care options and feel more confident in making their own healthcare choices rather than relying on family members [23, 24]. 

Individuals who have had close connections who engaged in end-of-life care planning are significantly more likely to perceive the preparation of an advance directive as important (Model 1). This strong positive association suggests that exposure to end-of-life planning within one’s social circle may heighten awareness and appreciation of the importance of making medical decisions in advance [25, 26].

## Discussion

This comparative study on advance directives among adults recruited in the U.S. and Taiwan revealed varying acceptance and awareness of advance directives. More importantly, it highlights the societal norms that may influence end-of-life decision-making. Based on previous studies, our study assumes cultural differences such as individualism in the U.S. and family collectivism in Taiwan. The primary focus was to investigate whether differences in beliefs and preferences regarding advance directives might indicate broader cultural influences. By analyzing attitudes toward decision-making, autonomy, and family involvement in medical choices, we aimed to explore how these cultural tendencies manifest in a specific healthcare context.

### Perceived importance of preparing an advance directive and willingness to discuss EOL care

We found that adults in Taiwan were not as reluctant to embrace advance directives as initially hypothesized, demonstrating greater openness than expected. After adjusting for confounding variables, the multivariable results indicate that participants in Taiwan are more likely than participants in the U.S. to believe in preparing an advance directive. This outcome is novel and surprising, especially since exposure to advance care planning and advance directives are relatively recent developments in Taiwan. Taiwan passed the Patient Right to Autonomy Act in 2016, which was then enacted in 2019 [13, 14]. This reflects a significantly shorter period of mainstream acceptance of advance directives in Taiwan compared to the U.S., where advance directives have been part of healthcare discourse for much longer, especially since the passage of the Patient Self-Determination Act in the 1990s [11, 12]. 

Recent legislative changes and public awareness campaigns in Taiwan may have heightened the public’s focus on advance directives, as reflected in our findings. In contrast, the more extended history of advance directives in the U.S. may have led to complacency or reduced public interest over time. Literature suggests that sustained engagement is essential for maintaining public awareness of end-of-life care, as the significance of advance directives can diminish without ongoing communication and reinforcement [27]. 

The recency effect, a type of cognitive bias, may further explain this difference observed [28, 29]. In Taiwan, recent legislative changes and public awareness campaigns around end-of-life care and advance directives remain prominent in the public’s memory, reinforcing their importance. Meanwhile, in the U.S., where discussions on advance directives began nearly 30 years ago, the initial urgency may have faded due to a lack of ongoing attention and public communication [27]. These findings suggest that Taiwan’s increasing emphasis on advance directives has played a critical role in the rapid adoption of advance directives despite their relatively recent introduction compared to the U.S.

### Willingness to let family and loved ones make health care or end-of-life care decisions during hospitalization for a serious illness

Another notable finding from this comparative study is that participants in Taiwan were significantly more willing to allow their family and loved ones to make health care or end-of-life care decisions during hospitalization for a serious illness (aOR = 1.73; 95% CI: 1.08–2.78, *p* < 0.05). This greater willingness to delegate healthcare care decisions to family members is likely influenced by cultural values deeply rooted in Taiwanese society, particularly those shaped by Confucian teachings [30]. 

In Taiwanese culture, filial piety—a Confucian concept emphasizing respect, obedience, and care for one’s parents and ancestors—is a fundamental societal value [31]. This cultural norm often leads to a greater reliance on family members to make critical medical treatment/care options for elderly relatives. The influence of filial piety creates a strong sense of responsibility among family members to act in the best interest of their elders, often taking on decision-making roles when individuals can no longer do so themselves. This deep-rooted cultural norm contrasts with the more individualistic values seen in American society, where personal autonomy and self-determination in healthcare decisions are prioritized [30]. The belief that family members are ideal decision-makers arises from their deep understanding of the family’s collective well-being, making them reliable proxies in such situations [32]. The higher willingness among participants in Taiwan to allow family members to make these healthcare decisions highlights a cultural tension between collective familial responsibility and individual autonomy. It suggests that approaches to advance care planning in Taiwan may need to consider and integrate these cultural values, where the role of the family is not merely advisory but integral to decision-making processes [13, 17, 19]. 

In contrast, American culture strongly emphasizes individual autonomy, particularly the right of individuals to make their own healthcare decisions, including end-of-life planning. This is reflected in the more widespread advocacy for individuals to personally complete advance directives in the U.S. and the focus on ensuring that their wishes are documented and honored. The reluctance of participants in the U.S. to have family members sign advance directives on their behalf may reflect a desire to maintain personal autonomy over their end-of-life care and ensure that their specific wishes are respected. Additionally, it may result from a reluctance to burden such a decision on family members.

### The belief that medical treatment/care options decisions between self and family would be consistent

The study uncovers a notable disparity in beliefs between adults in the U.S. and Taiwan regarding the alignment of *medical treatment/care options decisions* between themselves and their family members. Despite relying on family decision-making in Taiwan, adults in Taiwan were significantly less likely than American participants to believe that their medical treatment/care options would align with their family members’ decisions (aOR = 0.28). This reveals a significant and complex cultural tension between individual autonomy and family collectivism. In Taiwan, family members are often seen as extensions of the individual, prioritizing collective well-being over individual preferences. Therefore, in families where collective decision-making is the norm, individual preferences may be overshadowed by the perceived greater good of the family, leading to discrepancies in anticipated decisions. The inconsistency in medical treatment/care options between participants in Taiwan and their families may arise from generational differences, personal experiences, and varying familiarity with medical treatment/care options [13, 20, 23]. 

In contrast, American culture highly values individual autonomy and self-determination, especially regarding healthcare and end-of-life decisions [33–35]. The substituted judgment principle guides surrogate decision-making in the U.S. and posits that surrogates should act based on the patient’s wishes rather than their own [36]. These principles support our finding that American participants are more likely to believe their medical treatment/care options would be consistent with their families than the Taiwanese participants.

Taiwan could learn from the higher alignment of medical treatment/care options observed among American adults and their families. In Taiwan, efforts to enhance consistency in medical decisions may benefit from educational campaigns focused on medical care options and the significance of advance directives. In both societies, fostering such dialogue could bridge the gap between individual preferences and family intentions, leading to a more aligned and respectful decision-making process. Recognizing these cultural influences is essential for developing effective public health strategies and policies that honor diverse values related to end-of-life care.

### Social factors related to the attitudes toward advance directive beliefs and preferences

The findings highlight two social factors related to the attitudes toward advance directive beliefs and preferences in both countries. One is the education factor. Higher education is associated with a stronger preference for individual autonomy in healthcare decisions. This may indicate that individuals with higher levels of formal education place greater value on personal control over medical decisions. Consistent with existing literature [23, 24], education is crucial in shaping individuals’ attitudes toward medical decision-making. Those with higher educational attainment are often more aware of their rights as patients, have greater access to healthcare information, and may possess stronger critical thinking skills, enabling them to evaluate treatment options independently. As a result, they are more likely to prioritize personal agency in healthcare matters rather than relying on family members or medical professionals to make decisions on their behalf.

The second factor is social influence. This finding aligns with broader research indicating that social influence is a key driver of engagement in advance care planning (ACP) [25, 26]. When individuals witness family members or friends navigating end-of-life decisions—whether through creating their advance directives or making choices on behalf of loved ones—it can catalyze reflection and action. Such experiences may normalize discussions about future medical care, increase awareness of potential challenges in decision-making, and highlight the benefits of clearly documenting one’s preferences in advance. Furthermore, individuals who observe others engaging in end-of-life planning may gain a deeper understanding of the complexities involved in medical decision-making, reinforcing the need for proactive planning [25, 26]. 

### Limitations

Several limitations should be acknowledged. The participants recruited from the U.S. were primarily from New York State, limiting generalizability to other regions. To mitigate sampling bias, we matched a subset of the adult sample recruited in Taiwan (*n* = 186 out of 914) to the U.S. group by age. Secondly, data collection methods differed: the U.S. employed a hard-copy survey, while Taiwan used an online survey, which may have introduced response biases. We addressed this by using multivariate regressions to control for covariates and minimize bias in the results. Thirdly, both studies utilized the non-probability snowball sampling method, which depends on initial participants to recruit others. This approach often leads to a selection bias sample, as participants are likely to recruit others with similar characteristics, limiting the diversity and generalizability of the sample. In the New York State sample, 53% of participants held a graduate-level degree, compared to 12.1% of the broader U.S. population. Similarly, 30.25% were aged 30–59, versus 22.0% nationally [37]. A comparable pattern was observed in the Taiwan sample: 22.04% had a graduate-level degree compared to 8.2% of the general population, and 38.71% were aged 18–30, whereas this age group comprised only 17.1% of the general population in Taiwan [38]. While valuable for exploratory research, these limitations make it less ideal for drawing definitive conclusions. Fourthly, we recognize that the sample recruited from the U.S., mainly from New York State, represents a diverse population, making race an essential factor to consider. However, incorporating race/ethnicity into the regression models would be challenging due to its absence in the dataset recruited in Taiwan. Without parallel data from both samples, we could not meaningfully analyze race/ethnicity as a covariate while maintaining comparability. Lastly, unmeasured confounders may have influenced the findings. For example, chronic illness may increase ACP engagement [39], while healthier individuals may delay planning. Income level improves healthcare access and ACP participation, while lower-income poses barriers [40]. Religious faith may shape ACP preferences and engagement [41]. Future research needs to incorporate these factors to improve understanding of ACP disparities.

### Strengths

The manuscript has several strengths. It fills a key gap by comparing beliefs about advance directives between adults recruited from the U.S. and Taiwan. It offers insights into how cultural factors may shape end-of-life care preferences. Our study addresses a key gap in the literature by directly comparing beliefs and preferences toward advance directives between adults recruited in the U.S. and Taiwan. While previous research has examined advance care planning within individual cultural contexts, few studies have directly contrasted these perspectives to highlight the impact of cultural values and legal frameworks on advance directive adoption.

Our findings suggest that adults in Taiwan are more likely to value advance directives but also more hesitant about whether family decisions will align with their preferences. This underscores the need for policies encouraging structured family discussions and decision-making frameworks to reduce uncertainty and improve alignment between individual preferences and family expectations. Integrating family-centered communication strategies may help facilitate shared decision-making while respecting individual autonomy.

In the U.S., where familiarity with advance directives is higher but completion rates remain low, our findings highlight the need for renewed public engagement efforts to prevent complacency and increase utilization of advance care planning resources.

### Further research

Further research could delve deeper into cultural dynamics to design interventions that improve the acceptance and use of advance directives across diverse populations. Educational campaigns and legislative support in Taiwan can build on the growing openness to advance directives while encouraging family communication to align medical care option preferences better. In the U.S., efforts could focus on renewing public engagement, addressing complacency, and maintaining awareness of the importance of advance directives.

**Table 1:** Characteristics of the respondents (*N* = 348)

Variables	US (*N* = 162)		Taiwan (*N* = 186)		Chi-square	*P* value
	*N*	%	*N*	%		
Gender						
Male	60	37.04	39	20.97	12.65	< 0.0001
Female	102	62.96	147	79.03		
Education level						
Junior high school and below	4	2.47	3	1.61	42.34	< 0.0001
High school and equivalents	21	12.96	24	12.90		
College	51	31.48	118	63.44		
Graduate school	86	53.09	41	22.04		
Marital status						
Single	63	38.89	86	46.24	9.72	0.045
Married	77	47.53	83	44.62		
Separate/divorced	12	7.41	10	5.38		
Windowed	10	6.17	7	3.76		
Age group						
Young adults (18–30)	53	32.72	72	38.71	2.67	0.260
Adults (31–60)	49	30.25	60	32.26		
Older adults (60+)	60	37.04	54	29.03		
Full-time employment: yes	94	58.02	92	49.46	2.51	0.068
People close to you have engaged in end-of-life care planning: yes	87	53.70	75	40.32	6.23	0.008
Perceived importance of preparing an advance directive: yes	129	79.63	166	89.25	6.20	0.011
Willingness to discuss end-of-life care: yes	147	90.74	183	98.39	10.32	0.001
Willingness to have family sign advance directive for self: yes	75	46.30	110	59.14	5.73	0.011
The belief that advance directive decisions would be consistent between self and family: yes	127	78.40	94	50.54	28.99	< 0.0001

Rounding differences to 100% are possible

**Table 2:** Multivariate logistic regression results

Variables	Model 1: Perceived importance of preparing an advance directive		Model 2: Willingness to discuss end-of-life care		Model 3: Willingness to let family and loved ones make health care or end-of-life care decisions during hospitalization for a serious illness		Model 4: The belief that medical treatment/care options decisions between self and family would be consistent	
	aOR	95% CI	aOR	95% CI	aOR	95% CI	aOR	95% CI
Taiwan (ref: U.S.)	2.55^**^	(1.27, 5.12)	7.75^**^	(2.03, 29.50)	1.73^*^	(1.08, 2.78)	0.28^***^	(0.16, 0.47)
Age group (ref: under 30)								
31–60	3.85^*^	(1.25, 11.79)	0.84	(0.15, 4.57)	0.93	(0.47, 1.85)	0.53	(0.25, 1.11)
61 and above	2.41	(0.69, 8.41)	0.62	(0.09, 4.30)	0.95	(0.41, 2.19)	0.97	(0.39, 2.43)
Male	0.58	(0.29, 1.15)	0.46	(0.16, 1.28)	1.36	(0.82, 2.25)	0.722	(0.42, 1.25)
Full-time employment (ref: no)								
Yes	0.95	(0.46, 1.96)	0.60	(0.18, 2.03)	1.49	(0.89, 2.50)	1.37	(0.775, 2.43)
Education (ref: High school and below)								
College	0.93	(0.29, 2.92)	0.61	(0.13, 2.98)	0.46^*^	(0.22, 0.93)	0.75	(0.36, 2.46)
Graduate School	0.83	(0.26, 2.06)	2.76	(0.51, 14.85)	0.32^**^	(0.15, 0.67)	0.63	(0.29, 1.37)
Marital status (ref: divorced/ separated/widow)								
Married	0.83	(0.25, 2.80)	1.35	(0.25, 7.28)	1.71	(0.86, 3.39)	1.72	(0.83, 3.54)
Single	0.60	(0.16, 2.22)	0.73	(0.10, 5.30)	1.47	(0.666, 3.26)	1.01	(0.44, 2.33)
People close to you have engaged in end-of-life care planning (ref: no)								
Yes	3.37^***^	(1.62, 7.04)	1.36	(0.47, 3.94)	0.95	(0.60, 1.49)	1.62	(1.00, 2.64)

*p < 0.05, **p < 0.01, ***p < 0.001

## Data Availability

Data is available on reasonable request.
